# Conversion to Glaucoma After Ocular Trauma in Pediatric Patients

**DOI:** 10.3390/vision9010005

**Published:** 2025-01-14

**Authors:** Nur Cardakli, Rujuta A. Gore, Courtney L. Kraus

**Affiliations:** 1 Wilmer Eye Institute, Johns Hopkins Hospital, Baltimore, MD 21287, USA; nur.cardakli@jhmi.edu (N.C.); rujuta.g@gmail.com (R.A.G.); 2 OSF Children’s Hospital of Illinois, Peoria, IL 61614, USA

**Keywords:** pediatric glaucoma, pediatric glaucoma suspect, ocular trauma, pediatric ocular trauma

## Abstract

Background: The outcomes of pediatric glaucoma suspects with a history of ocular trauma remains unknown; we describe the rate of conversion to glaucoma of this population of patients at a research-intensive academic center. Methods: We conducted a retrospective case series of pediatric patients with a history of open- or closed-globe trauma who were being monitored as pediatric glaucoma suspects at the Wilmer Eye Institute between 2005 and 2016. Results: A total of 62 eyes from 62 patients with a history of ocular trauma were identified with a median age at presentation of 9.7 years (7.8 years) and a median follow-up of 2.7 (5.8 years). There were 29 eyes (46.8%) with open-globe injuries and 33 eyes (53.2%) with closed-globe injuries. A higher proportion of eyes that sustained closed-globe injuries were started on topical therapy for persistent ocular hypertension than eyes that sustained open-globe injuries (36.4% versus 10.3%, X^2^ = 5.6976, *p* = 0.017). Five eyes (8.1%) developed glaucoma during the follow-up period, all of which had a history of closed-globe injury (15.2%, X^2^ = 4.7794, *p* = 0.029). Four eyes of these eyes underwent glaucoma-related surgical intervention. Most eyes (3/5, 60%) that went on to develop post-traumatic glaucoma had undergone additional and/or concurrent intraocular surgical interventions to address sequelae of ocular trauma, such as traumatic cataract and retinal detachment. Conclusions: All eyes that developed glaucomatous damage or underwent glaucoma-related surgical intervention in this cohort of patients with a history of ocular trauma were those that had sustained close-globe injuries. No eyes that sustained open-globe injury progressed to glaucoma.

## 1. Introduction

Ocular trauma is a significant cause of monocular morbidity in the pediatric population, with global estimates ranging between three to six million children under the age of 15 years being affected by ocular trauma annually [1]. As in adults, pediatric ocular trauma can present with a range of severity, with some injuries resulting in visually devastating complications, including retinal damage, formation of traumatic cataract, development of post-traumatic glaucoma, endophthalmitis, amblyopia, and strabismus after open-globe repair [2,3,4,5,6]. These complications can have lasting effects on a child’s visual acuity and visual potential, an element of profound importance in a child’s biological, social, and psychological development, especially for children within the critical period of visual development.

Deviations from normal levels of intraocular pressure (IOP) have been shown to occur shortly after trauma, with low IOP often associated with an open-globe injury prior to repair and elevated IOP often associated with hyphema, closed-globe injury, or post-surgical repair of an open-globe injury [7,8,9,10]. While many of these eyes will show improvement in IOP with the initiation of IOP-lowering medication and/or time, a subset will continue to show progressive damage characteristic of glaucoma and will require escalation of medical therapy and/or surgical intervention [9,10]. Few studies have evaluated the risk factors for and the glaucoma-related outcomes of pediatric patients who have sustained globe trauma. One factor that has been previously reported as related to the risk of development of post-traumatic glaucoma is the mechanism of injury, with higher rates of elevated IOP and need for glaucoma-related surgical intervention in pediatric patients who had sustained closed-globe injury as compared to open-globe injury [11]. Additionally, prior retrospective studies have shown high rates of filtering surgery intervention among pediatric patients with early onset of traumatic glaucoma within a month of closed-globe injury in nearly half of patients [12]. These prior studies have specifically reported on the outcomes of pediatric patients who have already developed either ocular hypertension after globe trauma and/or pediatric patients who have already developed post-traumatic glaucoma. There remains a paucity of literature regarding the outcomes of patients being monitored as glaucoma suspects after a history of globe trauma.

## 2. Materials and Methods

This study presents the findings of a sub-analysis of pediatric patients with a history of ocular trauma within a larger retrospective chart review of outcomes of pediatric glaucoma suspects reported by our group [13]. In brief, potential pediatric glaucoma suspects were examined at a single center, the Johns Hopkins Wilmer Eye Institute, between 1 January 2005 and 1 June 2016 and identified by use of ICD9/ICD10 codes for “glaucoma suspect” (H40.00, H40.01), “physiologic cupping” (H47.239), “ocular hypertension” (H40.05), “hyphema” (H364.41), “iridocyclitis NOS” (H364.3), and additional trauma-related codes [“unspecified open wound of eyeball (H871.9), “traumatic cataract, unspecified” (H366.20), and “contusion of eyeball” (H921.3)]. Eyes were defined as “pediatric glaucoma suspects” if they met either the criteria for pediatric glaucoma suspects due to ocular hypertension or suspicious optic disc appearance according to the criteria defined by the Childhood Glaucoma Research Network (CGRN) [elevated IOP > 21 mmHg on at least two separate occasions without evidence of IOP-related damage or cup-to-disc ratio [CDR] ≥ 0.5, asymmetry of CDR ≥ 0.2 between two eyes, notching or narrowing of the neuroretinal rim in the absence of ocular hypertension, or other signs of glaucomatous damage] [14]. Additionally, eyes that were considered glaucoma suspects based on history of cataract surgery, ocular trauma, or an ocular or a systemic disorder with known association with glaucoma, including history of ocular trauma that otherwise did not fit the CGRN criteria for a glaucoma suspect at the initial visit, were also included. Eyes with a history of ocular trauma, age less than 18 years at initial presentation after trauma, and at least 180 days of follow-up after ocular trauma were included in the present study. The Institutional Review Board (IRB) of the Johns Hopkins University approved the study (protocol code IRB00110220, approved 25 January 2017), and the research abided by the Declaration of Helsinki.

Clinical data were extracted from the electronic medical record, including demographic data (age, sex, and race), information about the traumatic incident (mechanism of trauma, type of trauma, location of trauma, and complications from trauma), information about the first “initial” presentation after the traumatic injury (VA, IOP, vertical cup-to-disc ratio [“CDR”], central corneal thickness, corneal diameter, and gonioscopy findings), and information about the clinical course (subsequent change in IOP, changes in CDR, initiation of topical and/or oral IOP-lowering medications, whether surgery was performed to address the trauma and/or complications that arose secondary to trauma and the types of surgeries, and subsequent visual field and/or OCT testing findings when available). Due to the very small number of cases with central corneal thickness and corneal diameter measurements, as well as visual field and OCT testing performed, these data were not included in further reporting and analysis of the data.

The type of trauma was classified according to the Birmingham Eye Trauma Terminology (BETT) [15]. Eyes with open-globe injuries were identified as having sustained a penetrating laceration, perforating laceration, or globe rupture; eyes with closed-globe injuries were identified as having sustained a contusion or lamellar laceration. In situations in which further information about the type of trauma could not be ascertained from the electronic medical record, the term “unspecified blunt trauma” or “unspecified open-globe trauma” was used to describe those cases with closed-globe and open-globe injuries, respectively. The complications from ocular trauma recorded from the medical record comprised hyphema, traumatic cataract, iris damage (iridodialysis or iris sphincter damage), commotio retina, retinal detachment or tear, traumatic iritis, vitreous hemorrhage, choroidal rupture, orbital fracture, intraocular foreign body, endophthalmitis, angle recession, and development of glaucoma. Complications were noted if they occurred at any time in the follow-up period after the presenting trauma. The zone of injury was noted for eyes that had sustained open-globe traumatic injury in accordance with the classifications put forth by the Ocular Trauma Classification Group [16].

Several methods were used for IOP measurements, including applanation tonometry, Tonopen (Reichert, Inc., Depew, NY, USA) and iCare tonometer (Icare USA, Raleigh, NC, USA), either in clinic or in the operating room with measurements taken immediately upon induction with general anesthesia. Several clinicians had recorded “normal” or “soft” on palpation as an approximate measure for IOP; these findings were recorded but excluded from further quantitative analysis. Documented reasons for tonometry not being performed on these patients included lack of patient cooperation and concern for applying pressure to the globe in the setting of an acute open-globe injury.

### 2.1. Outcomes

The primary outcome measure was conversion to glaucoma, as defined by CGRN criteria with at least two of the following: elevated IOP (>21 mm of Hg), progressive increase in CDR, cup–disc asymmetry of ≥0.2 between eyes, focal optic nerve rim thinning, progressive increase in axial length or myopic shift outside normal limits, increased corneal diameter or presence of Haab striae, and visual field defect consistent with glaucoma [14]. Eyes that underwent a glaucoma-related surgical intervention prior to meeting the above-mentioned criteria for “childhood glaucoma” were also considered conversion to glaucoma. Initiation of IOP-lowering medication was a secondary outcome.

### 2.2. Statistical Methods

All numerical values are reported as mean (SD) when normally distributed or as median with interquartile range (IQR) for non-normally distributed variables. The Mann–Whitney U test was used to compare the median age at presentation and median length of follow-up between eyes that had sustained open- and closed-globe injuries. Student’s *t*-test was used to compare the mean IOP at initial and final presentations and mean CDR at initial and final presentations between eyes that had sustained open- and closed-globe injuries. Pearson’s chi-squared test was used to compare the distribution of gender, ancestry, eye laterality, proportion of eyes started on IOP-lowering medication, and proportion of eyes that converted to glaucoma between eyes that had sustained open- and closed-globe injuries. A *p*-value < 0.05 was considered statistically significant.

## 3. Results

Among the 1375 eyes being followed as pediatric glaucoma suspects in the Pediatric Ophthalmology division of the Wilmer Eye Institute, 62 eyes (4.5%) in 62 unique pediatric patients were identified as having a history of ocular trauma: a total of 29 eyes (46.8%) had sustained open-globe injuries, and 33 eyes (53.2%) had sustained closed-globe injuries with a median age at presentation of 9.7 years (IQR 7.8 years) and median follow-up time of 2.7 (5.8 years). Individuals were predominantly male (67.7%) and White (58.1%). As shown in Table 1, the demographics were similar between eyes that had sustained open- and closed-globe injuries, apart from the distribution of sex-affected (X^2^ = 6.397, *p* = 0.011) and a larger mean CDR at initial presentation in eyes that had sustained closed-globe injuries (*p* = 0.019).

### 3.1. Mechanism and Location of Trauma

Among eyes that had sustained open-globe injuries, 25 eyes (86.2%) sustained penetrating lacerations, 2 eyes (6.9%) sustained perforating lacerations, and 2 eyes (6.9%) sustained globe rupture. Over half of the injuries occurred at Zone 1 (17 eyes, 58.6%), followed by 5 eyes (17.2%) with injuries at Zone 2 and 3 eyes (10.3%) with injuries at Zone 3. There were four eyes (13.8%) without the location of trauma specified in the electronic medical record. The most common causes of open-globe trauma were a pencil (four eyes, 13.8%), a glass piece (three eyes, 10.3%), and a wooden stick (three eyes, 10.3%).

Among eyes that had sustained closed-globe injuries, 24 eyes (72.7%) sustained contusions, 7 eyes (21.2%) lamellar lacerations, and 2 eyes (6.1%) unspecified blunt injuries. The most common causes of closed-globe injury were a thrown rock (four eyes, 12.1%), a paintball (three eyes, 9.1%), and a BB pellet (three eyes, 9.1%).

### 3.2. Post-Traumatic Ocular Complications

**Figure 1:**
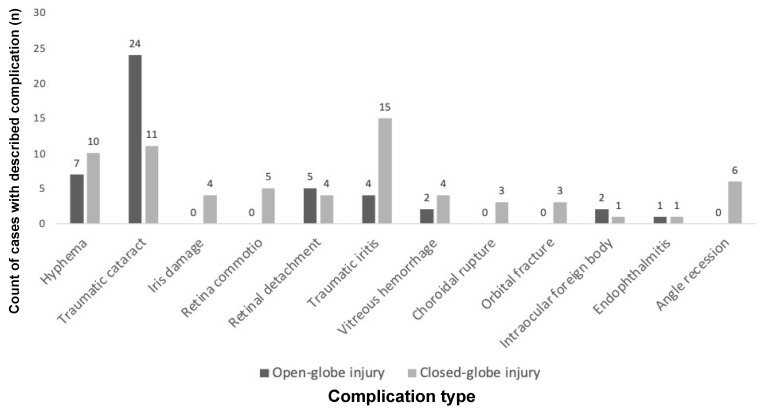
Counts of ocular complications after open-globe and closed-globe injuries. The most common complications after open-globe injury were traumatic cataract (24/29, 82.8%), hyphema (7/29, 24.1%), and retinal detachment (5/29, 17.2%). The most common complications after closed-globe injury were traumatic iritis (15/33, 45.5%), traumatic cataract (11/33, 33.3%), and hyphema (10/33, 30.3%).

As shown in Figure 1, the most common complications after open-globe injury were traumatic cataract (24/29, 82.8%), hyphema (7/29, 24.1%), and retinal detachment or tear (5/29, 17.2%). The most common complications after closed-globe injury were traumatic iritis (15/33, 45.5%), traumatic cataract (11/33, 33.3%), and hyphema (10/33, 30.3%). Gonioscopy findings were recorded in the electronic medical record for eight eyes.

Among the 24 eyes with traumatic cataract after open-globe injury, 19 eyes underwent lensectomy and were left aphakic. Of these, eight eyes later underwent secondary intraocular lens (IOL) placement (five sulcus IOLs, two iris-sutured IOLs, and one anterior chamber IOL), two eyes underwent lensectomy with primary IOL placement in the sulcus, and three eyes had lens material expulsed during the traumatic injury and were left aphakic.

Among the 11 eyes with traumatic cataract after closed-globe injury, 6 eyes underwent lensectomy and were left aphakic. Of these, four eyes later underwent secondary IOL placement (two sulcus IOLs and two iris-sutured IOLs), four eyes underwent lensectomy with primary IOL implantation in the posterior chamber (one in the sulcus, two in the capsular bag, and one unspecified), and one eye was monitored without surgical intervention.

### 3.3. Initiation of IOP-Lowering Therapy and Conversion to Glaucoma

Twelve eyes (36.4%) that sustained closed-globe injuries were started on eyedrops for persistently elevated IOP during the follow-up period, as compared to three eyes (10.3%) that had sustained open-globe injuries (X^2^ = 5.698, *p* = 0.017).

Among the three eyes with open-globe injuries that were started on IOP-lowering therapy, all three eyes were started on at least three, and up to five, classes of topical and/or oral IOP-lowering medications. Two of these eyes were started on medication upon initial presentation, and the third was started on IOP-lowering medication for post-operative pressure elevation. Among the 12 eyes with closed-globe injuries that were started on IOP-lowering therapy, 5 eyes were started on one class of topical medications, 4 eyes were started on two classes, 2 eyes were started on three classes, and 1 eye was started on five classes, including an oral carbonic anhydrase inhibitor. Ten of these eyes were started on drops at the time of initial presentation, and the remaining two were started on drops within the month after initial injury. None of the fellow eyes developed ocular hypertension or glaucoma during the follow-up period.

Five eyes (8.1%) ultimately developed glaucoma during the follow-up period with a median time to conversion to glaucoma of 8.5 months (IQR: 6.4 months to 10.2 years, range: 4 months to 15 years); all five eyes were those with closed-globe injuries (5/33 versus 0/29, 15.2% versus 0%, X^2^ = 4.779, *p* = 0.029). Four of these eyes underwent glaucoma-related surgical intervention. These outcomes of rates of IOP elevation requiring pharmacological treatment, conversion to glaucoma, and rate of surgical intervention are summarized in Table 2. Three eyes met the criteria for conversion to glaucoma based upon glaucoma-related surgical intervention, and two eyes met criteria for conversion to glaucoma based upon a progressive, asymmetric increase in CDR in the setting of continued ocular hypertension. Only one of these eyes was documented as having angle recession on gonioscopy; however, gonioscopic findings were not recorded for the other four eyes. Three of these eyes developed traumatic cataract, underwent lensectomy as well as other surgical interventions, and were left aphakic. Two of these eyes later underwent placement of secondary iris-sutured IOLs, after which ocular hypertension occurred; the remaining eye developed ocular hypertension while aphakic after lensectomy and vitrectomy. Notably, among the remaining 28 eyes with a history of closed-globe injury that did not ultimately convert to glaucoma, 13 eyes (46.4%) underwent other intraocular surgery. Of these, nine eyes underwent lensectomy (five had primary IOL placement; three were left aphakic, and one was initially left aphakic and later underwent secondary IOL placement), four eyes underwent pars plana vitrectomy (two combined with lensectomy), and two eyes underwent anterior chamber washout.

### 3.4. Brief Details About the Clinical Course of These Five Patients

1. An infant sustained unspecified blunt trauma with an unknown implement to the eye causing traumatic cataract. She underwent lensectomy shortly after initial presentation and was left aphakic. A secondary iris-sutured IOL was placed in the posterior chamber alongside a penetrating keratoplasty that was performed 14 years after the trauma, after which she developed ocular hypertension. She underwent a trabeculectomy at an outside hospital one year later.
2. A young child presented with a lamellar laceration after being hit in the eye with a rock, complicated by traumatic cataract and hyphema. He underwent several ocular surgeries over the five years post-trauma, including lensectomy, secondary iris-sutured IOL placement, IOL repositioning, and removal of a dislocated IOL. He eventually presented with a chronic closed-funnel retinal detachment after being lost to follow-up for three years. He underwent transscleral cyclophotocoagulation 10 years after the initial trauma for IOP above 40 mmHg.
3. A teenager who presented two years after sustaining a contusion after an assault who had not previously sought care and was found to have a traumatic cataract and retinal detachment. He underwent lensectomy with pars plana vitrectomy for retinal detachment repair and was initially left aphakic. He was started on IOP-lowering medications one month postoperatively for IOP > 30 mmHg. Despite maximum tolerated medical therapy, IOP continued to increase to above 50 mmHg with associated CDR change over the next two months, and he underwent tube shunt implantation and transscleral cyclophotocoagulation shortly thereafter.
4. A teenager sustained a contusion after a soccer ball to the face, leading to commotio retinae, a traumatic macular hole, and angle recession. Given elevated IOP, he was started on two classes of IOP-lowering medication immediately after trauma, which did not improve the IOP, and he ultimately underwent a tube shunt implantation within six months of the initial trauma.
5. A teenager sustained a lamellar laceration from a potato gun, complicated by hyphema, retina commotio, an orbital floor fracture, and lid laceration. He was started on two classes of IOP-lowering medications upon initial presentation for IOP of 30 mmHg. Nine months after the initial trauma, his CDR had progressed in the setting of persistent ocular hypertension after running out of IOP-lowering medications.

At the last follow-up visit, the mean IOP for eyes that had sustained closed-globe injury was higher than that of eyes that had sustained open-globe injury (19.9 ± 9.8 mmHg versus 14.7 ± 4.4 mmHg, *p* = 0.013). The mean CDR at the last follow-up visit was also larger for eyes that had sustained closed-globe injuries than those with open-globe injuries (0.3 ± 0.2 versus 0.2 ± 0.1, *p* = 0.009).

## 4. Discussion

The reported incidence rates of post-traumatic glaucoma in both pediatric and adult populations vary substantially in the literature, due in part to a lack of a consistent definition of “glaucoma” used as an endpoint. Whereas prior studies regarding post-traumatic ocular hypertension and/or glaucoma in pediatric patients have typically focused on subsets of patients who have already developed ocular hypertension and/or glaucoma [11,12], the present study is unique in following the outcomes of pediatric patients being monitoring as glaucoma suspects after ocular trauma and utilizes the CGRN criteria for conversion to “childhood glaucoma”, which to our knowledge has not been previously utilized for the standardization of nomenclature among pediatric patients with post-traumatic glaucoma or those being monitored as glaucoma suspects [14].

In this retrospective review of pediatric glaucoma suspects being monitored after ocular trauma, all eyes that developed glaucomatous damage or underwent glaucoma-related surgical intervention were those that had sustained close-globe injuries as opposed to open-globe injuries. Among eyes that had sustained a closed-globe injury, 15.2% ultimately developed glaucoma over a median follow-up period of nearly three years, with 80% of those eyes requiring a glaucoma-related surgical intervention during the follow-up period. Similarly, a higher proportion of eyes that had sustained closed-globe injury required topical IOP-lowering therapy as compared to those that had sustained open-globe injuries. This trend of higher rates of elevated IOP and the need for surgical intervention in eyes that had sustained closed-globe versus open-globe has previously been reported by Kalamkar and Mukherjee, who reported in a retrospective review of 205 eyes with pediatric ocular trauma in rural central India that 20.6% of eyes that had sustained closed-globe injury and 8.3% of eyes that had sustained open-globe injury developed elevated IOP and a quarter of eyes that had sustained closed-globe injury required surgical management, as compared to no eyes that had sustained open-globe injury and were able to be managed medically [11].

Among the five eyes that developed post-traumatic glaucoma after closed-globe injury in the present study, a sizeable proportion of these eyes developed ocular hypertension and traumatic glaucoma after other intraocular surgical interventions for complications of the traumatic injury, including lensectomy, penetrating keratoplasty, secondary intraocular lens placement and repositioning, and pars plana vitrectomy. Given the small number of eyes that developed glaucoma, it is difficult to parse out the relative contribution for risk of the development of glaucoma from the traumatic closed-globe injury itself versus from the effects of additional surgery. Reports linking cataract surgery to glaucoma risk have suggested that an age less than six months remains the greatest risk; thankfully most pediatric globe trauma patients are outside this higher risk group [17,18,19,20]. Among penetrating keratoplasty, an indication of trauma has been shown to be a risk factor for glaucoma [21]. It is important to note that none of the several eyes with open-globe injuries that underwent multiple other intraocular surgeries, including lensectomy, met criteria for the development of glaucoma during the follow-up period. The development of post-traumatic glaucoma in these pediatric eyes after closed-globe injury is likely multifactorial with a component of susceptibility owing to the mechanism of trauma, likely with contributions from steroid response and surgical inflammation in a damaged angle.

A recent study from the Pediatric Eye Disease Investigator Group (PEDIG) showed a 6% rate of glaucoma among children within 15 months of undergoing lensectomy for traumatic cataract [22]. Most eyes in the PEDIG study underwent primary IOL implantation (80%); most eyes did not have concomitant surgical procedures (66%), and the mechanism of ocular trauma—open- versus closed-globe injury—was not specified. All eyes in the PEDIG study that developed glaucoma were those that had undergone primary IOL implantation. In comparison, 3 eyes (8.8%) in the present study developed glaucoma over a median follow-up of 5.0 years among the 34 eyes that underwent lensectomy for traumatic cataract. All three eyes had sustained closed-globe injury and undergone several intraocular surgeries, including penetrating keratoplasty, pars plana vitrectomy, IOL repositioning, and removal of dislocated IOL. The higher rate of development of glaucoma in patients after lensectomy for traumatic cataract in the present study may represent a greater cumulative rate of the development of glaucoma over a longer period of follow-up, the effects of more severe traumatic injury, and/or the effects of several surgical interventions being performed.

An important structural factor attributed to one of the many mechanisms of post-traumatic glaucoma is the presence of angle recession. In a study of 40 teenagers and young adults who developed chronically elevated IOP post-trauma, 93% of eyes had varying degrees of angle recession on gonioscopy [23] although angle recession of at least 180 degrees was not found to be a significant predictor of late ocular hypertension (28+ days after injury) in a more recent study of pediatric patients who sustained traumatic hyphema after closed-globe injury [8]. Notably, only a minority of patients in the present study had gonioscopic findings reported in the medical record, presumably related to either a true or perceived inability for children to cooperate with gonioscopy, and only one of the eight eyes that had gonioscopy performed in the present study showed evidence of angle recession. Given the literature surrounding the elevated risk of post-traumatic glaucoma in eyes with angle recession, efforts should be made to attempt gonioscopy on all children post-trauma, especially after cases of closed-globe injury, for risk stratification.

A key deviation from the CGRN grading system for conversion to “childhood glaucoma” was the inclusion of glaucoma-related surgical intervention as an alternative endpoint for conversion to glaucoma, even if the specific criteria for “conversion to glaucoma” based upon CGRN criteria were not met. As the CGRN definition for conversion to glaucoma was defined as “IOP related damage to the eye”, these eyes were felt to be imminently at risk of permanent, glaucomatous damage without timely surgical intervention. Notably, surgical intervention has also been utilized as an equivalent endpoint for the development of childhood glaucoma in several other studies in the pediatric glaucoma literature [17,18].

There are several limitations worth noting. Several important endpoints in our study, including the initiation of IOP-lowering medication, decision to proceed to glaucoma-related surgical intervention, and decision to perform and interval between examinations under anesthesia were based on clinical discretion. As the clinicians in the present study represent a large group of pediatric ophthalmologists, glaucoma specialists, and ocular trauma surgeons, there is likely variability in the thresholds for each individual clinician to proceed with initiation of IOP-lowering therapy and to proceed with glaucoma-related surgical intervention, impacting several important endpoints in the present study. Cases for inclusion were identified by specific ICD9/ICD10 codes, which may have missed additional potential cases of pediatric ocular trauma if they were coded differently. Despite including over 10 years of pediatric glaucoma suspects being monitored at a large, designated eye trauma center, there was a small number of patients with a history of ocular trauma who met the inclusion criteria and an even smaller number of eyes in the present study that ultimately progressed to post-traumatic glaucoma, precluding the ability to perform additional analyses studying patient- and injury-specific factors. By nature of being a large academic referral center, patients with less severe injury or less complex post-traumatic course may have presented for initial consultation and early post-traumatic follow-up but may have been followed long-term by an outside provider and thus excluded from the study, impacting the cohort-wide severity of trauma and outcomes reported in the present study. Additionally, due to the nature of cessation of data collection after patients met the criteria for “conversion” to glaucoma, the present study likely underpredicts the true rate of glaucoma-related surgical intervention in this population who may have undergone glaucoma-related surgical procedures during their ongoing ophthalmologic care of their traumatic glaucoma.

In the present study, among this retrospective cohort of 62 pediatric patients being monitored as glaucoma suspects after a history of globe injury, over one in seven eyes that had sustained closed-globe injury went on to develop glaucomatous damage and/or undergo glaucoma-related surgical intervention over a median follow-up of nearly three years. In contrast, no eyes with a history of open-globe injury progressed to develop glaucoma. As evident both from the present study and from the body of literature surrounding ocular trauma in pediatric populations, pediatric ocular trauma may be accompanied by visually significant and devastating outcomes due to the trauma itself and associated complications. While careful evaluation at the time of trauma and diligent monitoring in subsequent years is crucial in identifying and managing the sequelae of ocular trauma, further emphasis must be placed on the role of primary injury prevention at a population level to reduce these visually devastating events from occurring in the first place.

## Figures and Tables

**Table 1:** Table of demographics stratified by type of ocular trauma.

	All Eyes	Open-Globe Injury	Closed-Globe Injury	*p*
Total eyes	62	29	33	
Sex—*n* (%) *				0.011
Female	20 (32.3)	14 (48.3)	6 (18.2)	
Male	42 (67.7)	15 (51.7)	27 (81.8)	
Race—*n* (%)				0.151
White	36 (58.1)	18 (62.1)	18 (54.5)	
Black	17 (27.4)	8 (27.6)	9 (27.3)	
Asian	2 (3.2)	0 (0)	2 (6.1)	
Hispanic	5 (8.1)	4 (13.8)	1 (3.0)	
Other/Multiple	3 (4.8)	0 (0)	3 (9.1)	
Median age at presentation (IQR)	9.7 years (7.8 years)	7.9 years (7.4 years)	12.3 years (7.4 years)	0.147
Eye laterality (R eye)—*n* (%)	28 (45.2)	16 (55.2)	12 (36.4)	0.138
Mean IOP at first presentation (SD)	19.0 mmHg (11.2 mmHg)	17.1 mmHg (12.2 mmHg)	21.0 mmHg (10.3 mmHg)	0.208
Mean CDR at first presentation (SD) *	0.3 (0.1)	0.2 (0.1)	0.3 (0.1)	0.019
Median length of follow-up (IQR)	2.7 years (5.8 years)	3.7 years (6.0 years)	2.1 years (5.2 years)	0.818

An asterisk (*) denotes categories in which there was a significant difference in the specified value between eyes with open-globe injury and eyes with closed-globe injury.

**Table 2:** Summary of key outcome measures stratified by type of ocular trauma.

	All Eyes	Open-Globe Injury	Closed-Globe Injury	*p*
Total eyes	62	29	33	
Eyes with elevated IOP requiring pharmacologic treatment (%) *	15 (24.2)	3 (10.3)	12 (36.4)	0.017
Eyes meeting criteria for conversion to glaucoma (%) *	5 (8.1)	0 (0)	5 (15.2)	0.029
Eyes requiring surgical intervention for glaucoma	4 (6.5)	0 (0)	4 (12.1)	0.053

An asterisk (*) denotes categories in which there was a significant difference in the specified value between eyes with open-globe injury and eyes with closed-globe injury.

## Data Availability

The data presented in this study are available on request from the corresponding author and are not publicly available due to privacy or ethical restrictions.
